# Do green bonds offer a diversification opportunity during COVID-19?—an empirical evidence from energy, crypto, and carbon markets

**DOI:** 10.1007/s11356-022-22492-0

**Published:** 2022-08-31

**Authors:** Miklesh Prasad Yadav, Satish Kumar, Deepraj Mukherjee, Purnima Rao

**Affiliations:** 1 Amity University, Noida, India; 2 Department of Management Studies, Malviya National Institute of Technology Jaipur, Jaipur, India; 3 Department of Economics, Ambassador Crawford College of Business and Entrepreneurship, Kent State University, Kent, OH 44240 USA; 4 Fortune Institute of International Business, New Delhi, Delhi, 110057 India

**Keywords:** Green bond, Energy market, Cryptocurrency, Carbon market, Connectedness, Diversification

## Abstract

The present study is a novel attempt to unravel the connectedness of the green bond with energy, crypto, and carbon markets using the S&P green bond index (RSPGB). We consider MAC global solar energy index (RMGS) and ISE global wind energy index (RIGW) as proxies of the energy market and use bitcoin and the European energy exchange carbon index (REEX) for the cryptocurrency and carbon market. Employing the Diebold and Yilmaz (2012), Baruník and Krehlík (2018), and wavelet coherence econometric techniques, we find that the energy market (RMGS) has the highest connectedness derived from other asset classes, and bitcoin (RBTC) has the least connectedness. Concurrently, we find that the risk transmission is heterogeneous in different scales as the short period has less connectedness than the medium and long run. We conclude that the overall diversification opportunity among green bonds, energy stock, bitcoin, and the carbon market is more in the short-run than in the medium and long-run. In summary, our findings on the green bond market will provide investors, portfolio managers, and policymakers with critical insight into ensuring a sustainable financial market.

## Introduction

Risk is inevitable in investment (Rao et al. 2021). According to Lopes (1987), “risk refers to situations in which a decision is made whose consequences depend on the outcomes of future events having known probabilities.” Portfolio diversification brace risk mitigation to a larger extent (Lassance et al. 2022; Zaimovic et al. 2021; Sharma et al. 2020). The advent of globalization and digitization has dismantled the distinct separation between different financial markets. It has resulted in close interconnectedness and dependencies between the markets and their activities (Bernardi and Petrella 2015). This century has witnessed shocks in economic crises, pandemics, sudden climate changes, wars, etc. According to International Monetary Fund (2020), climate change can lead to capital loss to economic actors and disruption in economic activities.

Further supported by Guo et al. (2021), it will take away 10% of global wealth by 2050. Thus, these common frequent, dynamic global changes pose a high risk against the investors’ return expectations. These events/changes have made risk interdependence more observant and visible. It further accentuates the spillover effect, a key factor in spreading the systemic risk. Hence, examining the interconnectedness among various investment alternatives is much required for portfolio risk management.

Recent literature on investment has documented the attention of investors toward green investments (Liu et al. 2022; Ren et al. 2022b; Pham and Huynh 2020; Doval and Negulescu 2014). Low carbon investments support building resilient infrastructure and stimulate sustainability. This is in agreement with the Sustainable Development Goals (SDGs) 2030. According to Busch et al. (2016), financial markets have the capacity in promoting sustainable development through energy-efficient investments. Unequivocally, there is a pressing requirement to facilitate the direction as well as the magnitude of investments toward low carbon and green stocks (Louche et al. 2019). Therefore, financial markets probably are one of the key drivers in leading the shift toward low-carbon economies. Further, investors and firms have already been exposed to considerable risk regarding their investments in conventional securities. Thus, it calls for innovative ways of assimilating climate change-related financial risk for investors. The issuance of green bonds is one of the steps toward funding future green projects and restructuring the old projects to aid in decarbonating and moving toward a carbon-neutral economy. When the World Bank issued its first green bonds in 2008, they sparked investors’ attention as a fixed-income vehicle. After that, the market of global green bonds rose from 11 billion USD in 2013 to 259 billion USD in 2019, representing an increase of 51% over 2018 (Climate Bonds Initiative 2018). The green bond market accounts for just more than 1% of the global bond market. Most (38%) of the revenues were employed in the power industry. It can be said that the green bond market is tiny but rising quickly.

In recent times, cryptocurrency, especially bitcoin, has also gained much attention for its safe-haven role and potential hedging, which poses tremendous opportunities for policymakers (Abakah et al. 2022; Juškaitė and Gudelytė-Žilinskienė 2022; Sukumaran et al. 2022; Yan et al. 2022b). The cryptocurrency market recorded a huge market capitalization, nearly about $386.2 billion U.S. dollars, by June 2022^1^. Meanwhile, a study indicates that cryptocurrency is still ambiguous to diversify in financial markets as it is considered technology securities or financial bubbles. Additionally, due to the requirement of huge power backup for the algorithm of bitcoin transactions, it is an alarming issue for the environment as it is one of the reasons for carbon emission (Naeem et al. 2021). Investors must engage in environmentally sound projects and diversify their portfolios to minimize risk against the crypto and carbon market. Therefore, investors need to check the dynamic linkages among various investment alternatives to benefit from a low correlation risk-return trade-off between the assets. This paper attempts to unravel the spillover from the green bond to energy, cryptocurrency, and carbon markets to check the diversification opportunity.

The remainder of this paper is structured as follows: “Research motivation” section provides research motivation, followed by extensive literature based on the spillover of green bonds with other markets. The “Data and econometric models” section contains data description and econometric models. Further, “Empirical results and discussion” section furnishes empirical results and discussion, followed by the conclusion and policy implication presented in “Conclusion and policy implications” session.

## Research motivation

Sustainable development is one of the most important goals that economies must achieve at the present time (Kihombo et al. 2021; Ahmed et al. 2022). Environmental sustainability is necessary for future growth and development. It is the joint responsibility of all stakeholders to align strategies that can create a substantial impact in maintaining ecological footprints (Caglar et al. 2022). Certainly, the adverse climate change is a pressing concern and the world is visibly witnessing severe damage to the ecosystem. This ultimately impedes the economic growth of the nations. To promote sustainable business practices, current, as well as future investments, should be directed toward energy-efficient projects which involve low carbon emissions and more dependency on renewable resources. This will also help in accomplishing SDG 2030. Therefore, current and future research should be focused on integrating investments with sustainability. As it will not only support the ecosystem but will also generate safe returns for investors (Corbet et al. 2019; Mert and Caglar 2020).

Sustainable growth requires reducing carbon emissions, which is crucial for maintaining global temperature rise within acceptable boundaries that prevent climatic disasters like the disappearance of tiny islands and shore areas due to rising sea levels (Bachelet et al. 2019). Environmental pollution is most. These promises have concentrated on mitigation and reshaping the techniques, including green bond issuance, carbon pricing, and clean energy for tackling climate issues. Further, change in climate creates considerable portfolio investment risk, especially for the institutional investors having massive funds size for the management under them, while also presenting investment possibilities (Mercer 2015). As a result, worldwide agreements have been reiterated to reduce CO_2_ emissions and limit increasing temperature levels to combat climate change (Intergovernmental Panel on Climate Change 2014).

On the contrary, bitcoin is one of the leading cryptocurrencies, which raises the environmental concern as the algorithmic structure of bitcoin requires huge power backup to authenticate the transaction. In addition, the footprint (carbon) on one bitcoin transaction is compared to 37% million tons of carbon emission (CO_2_) of New Zeeland (Karim and Naeem 2021). Therefore, one asset class may affect another. It further opens doors for the research examining the interconnectedness and spillover effect between asset classes having low correlation and provides diversification opportunities to investors and fund managers. This paper investigates the dynamic connectedness of green bonds with the energy, crypto, and carbon market and contributes to the extant literature in three folds: first, the energy market has been measured by two different proxies, namely MAC global solar energy index (RMGS) and ISE global wind energy index (RIGW), which was not included earlier in any study together. This also implies robustness and establishes generalizability. Second, focuses on enriching the literature as very few studies are available on the spillover of green bonds with energy, crypto, and the carbon market. Third, methodological rigor is related to applying novel models like Diebold and Yilmaz (2012), Baruník and Krehlík (2018), and wavelet coherence to examine the dynamic spillover among constituent markets. The research further provides chances to design diversification strategies to fund managers and practitioners based on the empirical results illustrated by the research.

## Literature review

It has been central to examine the dynamic spillover from one market to another for portfolio diversification. Literature has shed light on dynamic spillover from the green bond to financial, commodity, crypto, energy, and other markets. There is evidence of dynamic linkage of various assets class in the studies of Tiwari et al. (2022), Liu et al. (2021), Le et al. (2021), Hammoudeh et al. (2020), Reboredo and Ugolini (2020), and Garrett-Peltier (2017). Further, the results derived from these studies are mixed. Reichelt (2010) emphasized the role played by green bonds in mobilizing private funds to mitigate climate change and argued that green bonds might be considered an initiative that confirms that financial markets can be used to support eco-friendly projects. However, public credits with AAA ratings might be employed to channel funding to mitigate and adapt the initiative are uncommon. They further emphasized the development of fixed-income instruments that optimize the trade-off between volume and credit for mobilizing the resources on the huge scale required to combat climate change. Pham (2016) studied the connection of green bonds with other financial markets employing the multivariate GARCH model to check the possibility of dynamic linkages and found that green bond volatility is transmitted to considered financial markets.

Similarly, Reboredo (2018) found that the green bond market has a poor co-movement with the equity and energy markets, providing significant diversification benefits to investors. Unfortunately, in the corporate and treasury market, the advantages of diversification are negligible to the investors. To support this, Reboredo and Ugoline (2020) further extended their work to highlight the direct and indirect exposures of events between the green bond and financial markets by using the structural VAR. The findings imply that green bonds are tightly linked to forex and fixed-income markets. Therefore, price spillovers from both markets are insignificant.

Ferrer et al. (2018) employed Baruník and Krehlík (2018) model to investigate the dynamic connectedness between the US clean energy stock and crude oil prices. It was found that both have a short-term linkage only, which infers that diversification is not possible in the short run. Further, Nguyen et al. (2021), by using the wavelet framework, demonstrated the time-frequency co-movement between green bonds and different assets class; they noted the presence of low and negative association of green bonds with different assets class due to which investors can park their money to mitigate the risk. Further, to investigate the role of green bonds as an effective hedging mechanism, Jin et al. (2020) considered green bonds as diversifying the carbon market risk. They employ DCC-GARCH, TGARCH, and GJRGARCH models and observed that the green bond index helps hedge the carbon futures. Similarly, academic interest is burgeoning in cryptocurrency as it plays a safe and potential hedging role in diversifying with other markets. In this line, Shahbaz and Sinha (2019), Giudici and Abu-Hashish (2019), and Baur et al. (2018) examined the connectedness between cryptocurrency (very often bitcoin) and other financial markets by employing a battery of tests and found that bitcoin is diversifier.

Various researchers analyze the dynamic spillover across green bonds and other asset classes. During the severe swings, Pham and Huynh (2020) found co-movement between the green bond and equity market. Kanamura (2020) examined the price correlations between the green bond and energy commodities using daily data from November 3, 2014, to December 31, 2018. It is found that MSCI and S&P green bonds are positively connected, whereas solactive bond is negatively connected with WTI and Brent crude oil. Liu et al. (2021) studied the dynamic linkage between green bonds and the clean energy market, considering daily observation from July 5, 2011, to February 24, 2020. They employed CoVAR and found that clean energy has an asymmetrical contagion influence on green bonds. While investing in various assets class, it is noticed that a green bond is assumed to be a shield and safe-haven asset for traditional equities, stable income, commodities, and forex securities (Arif et al. 2021).

In contrast, Antonakakis et al. (2020) found the spillover among S&P green bonds, MSCI global environment, Dow Jones sustainability indices, and S&P Global clean energy, which infers that risk cannot be mitigated. Similarly, Naeem et al. (2021) find asymmetric spillover among asset classes during a different time and frequency cycles. The positive return spillover is mostly visible in the short run, whereas the negative return spillover is in both the long and short run. Further, it explains green bonds as an aid against risk in another commodity market. Further, Nguyen et al. (2021) studied the relationship between green bonds and found that green bonds act as diversification opportunities due to the low correlation effect with other assets class.

A section of emerging literature furnishes green bonds as an investment alternative carrying fixed-income assets that hedge for various asset classes like renewable stocks, carbon emissions, and other markets. Sangiorgi and Schopohl (2021) surveyed asset managers on green bonds, especially in European countries. They observed that investors actively invest in the green bond market but prefer those green bonds issued by sovereign countries. It is also found that competitive pricing is one factor in asset managers’ choice of a green bond investment. The interest of investors is growing in a green bond, due to which the scale of growth in the green market is intriguing concerning green premium. On this note, MacAskill et al. (2020) undertook a study and encompassed whether there is “green premium” or “greenium” in green bond pricing. The consistent existence of green premium by 56% in the primary market while governments issue 70% of studies in the secondary market. We also consider the environmental impact of green bonds, which is one of the crucial components for financing green projects. In this line, Flammer (2021) strongly believes and argues the three-implication motives that companies are strengthening their image of philanthropy and being environmentally responsible by issuing and investing in green bonds. To check the hedging, Jin et al. (2020) emphasized the importance of green bonds and considered one of the effective hedging instruments for the carbon market. They computed three hedge ratios containing a battery of tests related to the transmission of dynamic linkages, for instance, symmetrical and asymmetrical dynamic conditional correlation. It is observed that the green bond index is a fair hedge for the carbon market, which is supported by Reboredo (2018) as he confirms the low correlation of green bonds with the energy market and finds diversification opportunities among these markets. Tiwari et al. (2022) conjecture a dynamic spillover among green bonds, renewable energy, and the carbon market. They employed TVP-VAR and LASSO dynamic connectedness based on daily observation and found that total connectedness among these assets class is heterogeneous.

Additionally, it signifies that the clean energy market dominates other markets. The studies in Table 1 emphasized green finance in the form of green bonds and its drivers. Additionally, it highlights the dynamic linkages of green bonds with asset classes like equity, energy, carbon, cryptocurrency, and other markets employing a battery of tests. To sum up, the existing studies, on the one hand, focus on the linkage or spillover from green bonds to various markets. On the other hand, a strand of literature is also found on the connectedness of green bonds with renewable energy and the carbon market. Still, there are a handful of studies on green bond linkage with energy, carbon, and cryptocurrency market, which builds the motivation of this research. Further, the literature is fragmented and lacks evidence regarding studying three different markets, which offer different diversification opportunities to investors.

**Table 1:** Recent literature published on green bonds and their interconnected ness with other financial assets

S.No.	Paper title	Author	Year	Purpose	Findings
1	Is There an Asymmetric Relationship between Economic Policy Uncertainty, Cryptocurrencies, and Global Green Bonds? Evidence from the United States of America	Syed et al.	Syed et al. 2022	The study investigates the asymmetric relationship between green bonds, U.S. economic policy uncertainty (EPU), and bitcoins by using the Nonlinear Autoregressive Distribution Lag	It documents that green bonds are not a different asset class, and they mirror the performance of other asset classes, such as clean energy, oil prices, and bitcoins
2	The interrelationship between the carbon market and the green bonds market: Evidence from wavelet quantile-on-quantile method	Ren et al.	Ren et al. 2022a	To enumerate the interrelationship between the carbon futures and green bond markets.	The study discovers the positive effects of the carbon futures in the medium to long term and intermittent performance in the short term. The effects are more distinct when both markets are in a risky state.
3	Driving green bond market through energy prices, gold prices and green energy stocks: evidence from a non-linear approach	Yan et al.	Yan et al. 2022a	To assess the factors that support the global green bond markets, such as energy prices, gold prices, and green energy stocks	The findings revealed that gold and energy prices have an inferior effect on the green bonds, the green energy stocks have a swelling effect on the green bonds market
4	Dynamic nonlinear connectedness between the green bonds, clean energy, and stock price: the impact of the COVID-19 pandemic	Chai et al.	Chai et al. 2022	To examine the dynamic nonlinear connectedness between the green bonds, clean energy, and stock price around the pandemic in the international markets	This study verifies the presence of nonlinear and dynamic correlation among green bonds, clean energy and stock prices
5	Impacts of COVID-19 outbreak, macroeconomic and financial stress factors on price spillovers among green bond	Mensi and Rehman	Mensi et al. 2022	To examine the influence of the pandemic and global risk factors on the upside and downside price spill overs of MSCI global, building, financial, industrial, and utility green bonds	The spill over index method shows noteworthy dynamic volatility spill overs that strengthen during the pandemic
6	Do green bonds de-risk investment in low-carbon stocks?	Reboredo et al.	Reboredo et al. 2022	To reconnoitre how green bonds could de-risk investments in low-carbon assets by considering diverse market conditions.	The research documents that green bonds have substantial diversification doles when they are incorporated in low-carbon investment portfolios
7	Extreme directional spillovers between investor attention and green bond markets	Pham and Cepni	Pham and Cepni 2022	To study how the spillovers between investor attention and green bond performance fluctuate across ordinary and risky market environments	Spillovers are time-varying, asymmetric, and expressively influenced by stock, oil, bond market volatility, and economic policy improbability.
8	Asymmetric connectedness between cryptocurrency environment attention index and green assets	Kamal and Hassan	Kamal and Hassan 2022	To analyze the impact of the cryptocurrency environment attention index (ICEA) on clean energy stocks and green bonds using a range of econometric methods.	COVID period reveals higher connectedness and changes in the direction of contagion among assets and a lack of significant relationship between ICEA and asset returns.
9	Dependence structure and dynamic connectedness between green bonds and financial markets: Fresh insights from time-frequency analysis before and during COVID-19 pandemic	Elsayed et al.	Elsayed et al. 2022	To examines the interdependence between green bonds and financial markets	The interconnection between green bonds and financial markets is capricious over time. This evidence provides suggestions for international investors regarding risk management and portfolio decisions.
10	Should investors include green bonds in their portfolios? Evidence for the USA and Europe	Han and Li	Han and Li 2022	To explore the role of green bonds in asset allocation	Portfolios with green bonds outpace portfolios with traditional bonds wrt risk-adjusted returns.

Table 1 summarizes the recent literature published on green bonds and their interconnectedness between different markets.

## Data and econometric models

### Data and preliminary analysis

This study attempts to unravel the dynamic spillover of the green bond with energy, cryptocurrency, and the carbon market. To represent the indicator of the green bond, S&P green bond index (RSPGB) is considered. In contrast, MAC global solar energy index (RMGS) and ISE global wind energy index (RIGW) measure the energy market. The MAC global solar energy index tracks the Invesco Solar exchange-traded funds (ETF) traded on the New York Stock Exchange. In contrast, the ISE global wind energy index tracks the company’s activity in the wind energy industry. The major reason to consider these energy indices is that these are modified float-adjusted market capitalization indexes. Further, bitcoin and the European energy exchange carbon index (REEX) are the proxies of cryptocurrency and the carbon market, respectively. These variables are considered by the following researchers, namely Hanif et al. (2021), Liu and Liu et al. (2021), and A.K Tiwari et al. (2022). The S&P Green Bond Index is designed to finance environmentally friendly projects (Tiwari et al. 2022). Bitcoin is considered a decentralized digital currency that intends to follow the ideas in the white paper.

At last, European Energy Exchange Carbon index is computed and presented as an exchange-based price for the market value of European Union emission allowances. The daily closing price of the constituent series from October 1, 2015, to December 13, 2021, from Bloomberg has been collected. The main reason for collecting the data from October 2015 is that the drive toward green finance was unleashed in 2015 by the Paris Climate Agreement in the form of green investment. Further, after December 2021, the impact of the COVID-19 outbreak has been seen less on constituent markets comparatively. The focus on renewable energy and other initiatives toward green finance started (ECLAC 2017). Further, the data is converted into log returns using ln(Pt)-ln(Pt-1). The description of variables is presented in Table 2.

**Table 2:** Data description of constituent variables

Asset/index	Proxy	Acronyms	Source
Green bond	S&P Green bond index	RSPGB	Bloomberg
Energy market	MAC global solar energy index	RMGS	
	ISE global wind energy index	RIGW	
Cryptocurrency	Bitcoin	RBT	
Carbon Market	European Energy Exchange Carbon index	REEX	

Source: authors’ presentation

Table 2 exhibits the descriptive statistics of return on the green bond, energy market, and carbon market. Considering the mean return, it is observed that the mean return of each series is positive; the mean return of bitcoin (RBTC) and the carbon market (REEX) is high (0.0015), followed by the energy market, namely RMGS (0.0007), RIGW (0.0004), and green bond (RSPGB) that is (0.0001, therefore, the green bond is witnessed with least average return. REEX is recorded with the highest standard deviation, indicating that the carbon market is riskier than other markets. In addition, each series is left-skewed and leptokurtic distributed, which confirms the asymmetrical pattern; the same has been confirmed by the Jarque-Bera test, which is statistically significant at a 1% significance level. This result is similar to Rbeordo (2018) and A.K. Tiwari et al. (2022). The series must be stationary to check the dynamic linkages; hence, we apply the augmented Dickey-Fuller (ADF) and Philips and Perron (P.P.) test. The result from both tests infers that all series are statistically significant at a 1% level and confirm the presence of stationarity. In addition, the Fourier unit root test developed by Enders and Lee (2012) is employed to check the stationarity. This test helps to encounter the series which has more structural breaks at unknown dates. The test statistics of each series (depicted in Table 3) are greater than the critical value. Hence, this test confirms the stationarity in constituent series. Furthermore, these markets exhibit the ARCH effect at a 1% significant level. On this note, our choice of models to examine the dynamic linkages is supported by summary statistics.

**Table 3:** Summary statistics of the green bond, energy, and carbon market

	RSPGB	RMGS	RIGW	RBTC	REEX
Minimum	− 0.0241	− 0.1496	− 0.1259	− 0.4973	− 0.9787
Maximum	0.0201	0.1132	0.0989	0.2034	1.1130
Mean	0.0001	0.0007	0.0004	0.0015	0.0015
St. dev	0.0030	0.0204	0.0113	0.0423	0.0957
Skewness	− 0.5936	− 0.5595	− 1.0627	− 1.0564	− 0.3040
Kurtosis	7.8513	6.5817	17.7326	14.2510	73.9348
Jarque-Bera test	0.0100**	0.0000***	0.0000***	0.0100***	0.0010***
ADF-test	0.0010***	0.0100**	0.0001***	0.0000***	0.0000***
PP Test	0.0000***	0.0000***	0.0000***	0.0000***	0.0000***
ARCH Test	0.0000***	0.0000***	0.0100**	0.0000***	0.0000***
Fourier Unit root test	− 5.3114**	− 4.8256**	− 4.7511**	− 5.0003**	− 11.8355***

** and *** indicates the significance level at 1% and 0.01% respectively

**Fig. 1:**
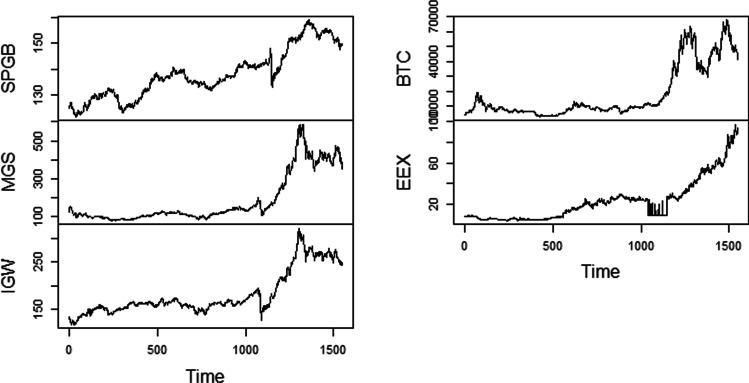
Time series plot of raw series

**Fig. 2:**
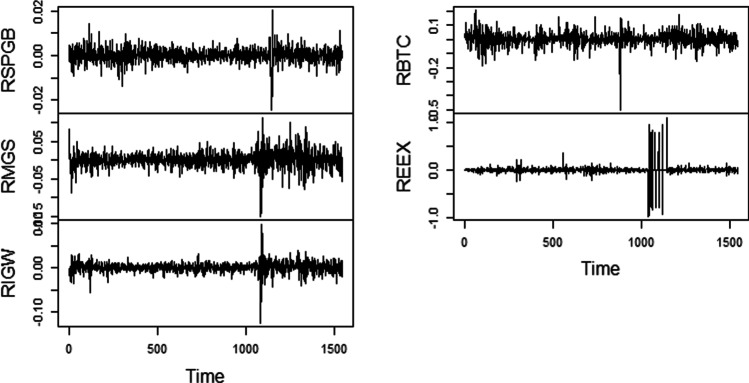
Time series plot of constituent return series

Figure 1 and 2 display the graphical representation of constituent series’ raw and log returns, respectively. Regarding Fig. 1, the intense fluctuations have been noticed in each asset class and have the presence of a stochastic trend. Similarly, the log return of each asset under examination is more pronounced in the COVID-19 pandemic. Further, volatility clustering is seen multiple times in each series, which depicts that high and low changes follow high changes followed by low changes (Sharma et al. 2020).

### Econometric models

In this paper, a battery of tests like Diebold and Yilmaz (2012), Baruník and Krehlík (2018), and the Wavelet coherence model has been employed to examine the dynamic spillover of green bond with energy, crypto, and carbon market. There is a great role in the magnitude of volatility and its connectedness over time and frequency in optimizing the fund allocation of investors’ assets. Additionally, the volatility of various assets class differs from each other (Ferrer et al. 2018). In this direction, decomposition of connectedness over time (short, medium, and long) and frequency is of utmost priority for the investors. Diebold and Yilmaz’s (2012) model assumes that connectedness remains the same over the short, medium and long time. Baruník and Krehlík (2018) model is applied to separate the connectedness over the period to overcome this problem. But these two models do not depict the lead-lag relationship of this volatility among constituent markets in various time and frequency domains. For the same, we employ the wavelet coherence model. A detailed understanding of these models is as below.

#### Diebold and Yilmaz (2012) and Baruník and Krehlík (2018)

The Diebold and Yilmaz (2012) model is based on variance decomposition analysis to check variables’ magnitude and dynamic linkages (Pesaran, M.H. and Shin, Y., 1998). It helps to examine the within, to, and fro transmission of information from one variable to another. In this model, the shocks of the variables can be presented through H-step forecast error variance decomposition that can be shown below:

1 $${d}_{ij}^{gH}=\frac{\sigma_{jj}^{-1}\sum_{h=0}^{H-1}{\left({e}_i^{\prime }{\varTheta}_h\sum {e}_j\right)}^2}{\sum_{h=0}^{H-1}\left({e}_i^{\prime }{\varTheta}_h\sum {\varTheta}_h^{\prime }{e}_i\right)}$$

where ${d}_{ij}^{gH}$is the H-step generalized variance decomposition (GVD) matrix, e_*j*_ is a selection vector, where *j*th element is unity and zero otherwise. *Θ*_*h*_ is the coefficient matrix in the infinite moving average representation of the non-orthogonalized VAR, Σ is the covariance matrix of the shock vector and ${\sigma}_{jj}^{-1}$ is its *j*th diagonal element. The generalized variance decomposition (GVD) matrix is normalized by the tow sums to obtain pairwise directional connectedness from *j* to *i.*

2 $$\widetilde{d_{ij}^g}=\frac{d_{ij}^g}{\sum_{j=1}^Nd_{ij}^g}$$

In this equation, $\overset{\sim }{D_{ij}^g}$is used to calculate the generalized connectedness and $\sum_{j=1}^N\overset{\sim }{d_{ij}^g}$=1 and $\sum_{i,j=1}^N\overset{\sim }{d_{ij}^g}=N$. The pairwise connectedness can be shown mathematically as below:

3 $${C}_{i\leftarrow j}^H={d}_{ij}^H$$

where ${C}_{i\leftarrow j}^H$ denotes the cross-spillover toward *i* due to shock in variable *j*. ${d}_{ij}^H$ denotes the *ij*th H-step forecast error variance, i.e., fraction of variable *i*'s H-step forecast error variance dur to shock in variable *j*. Since in general, ${C}_{i\leftarrow j}^H\ne {C}_{j\leftarrow i}^H$, there are *N*^2^ − *N* separate measures of pair-wise directional spillover. Thus, the net pairwise directional spillover is defined as:

4 $${C}_{ij}^H={C}_{j\leftarrow i}^H-{C}_{i\leftarrow j}^H$$

In Eq. 4, ${C}_{i\leftarrow j}^H$ denotes the cross-spillover toward *i* due to shock in variable *j* and ${C}_{j\leftarrow i}^H$ denotes the cross-spillover toward *j* due to shock in variable *i.* Next, it is followed by Baruník and Krehlík (2018) model to furnish the spillover or dynamic linkages of green bond with energy, crypto, and carbon market in various time horizon, for instance, short, medium, and long run. This model is different from Diebold and Yilmaz (2012) model in form of time horizon (Elsayed and Yarovaya 2019). To be precise, it decomposes the spillover obtained from D.Y. (2012) model in various frequencies based on spectral formulation of variance. This model can be expressed mathematically as follows:

5 $${\left(f\left(\omega \right)\right)}_{j,k}\equiv \frac{\sigma_{kk}^{-1}{\sum}_{h=0}^{\infty}\left({\left|\varPsi \left({e}^{- iw}\right)\varSigma \Big){}_{j,k}\right|}^2\right)}{\sum_{h=0}^{\infty }{\left(\varPsi \left({\omega}^{- iw}\right)\varSigma {\varPsi}^{\prime}\left({e}^{+ iw}\right)\right)}_{jj}}$$

In Eq. (5), *Ψ*(*e*^−*iw*^) is considered as impulse response Fourier transformation. The terminology (*f*(*ω*))_*j*, *k*_ represents jth variable band at frequency 𝜔 because of the disturbance in *k*th variable. Therefore, the coefficient of frequency is considered as denominator holding the *j*th variable spectrum, that is, the multi-spectral of the diagonal elements of the density of x_i_ at any given frequency 𝜔. Additionally, the weighting function can be computed as follows:

6 $${\varGamma}_j\left(\omega \right)=\frac{{\left(\varPsi \left({\omega}^{- iw}\right)\varSigma {\varPsi}^{\prime}\left({e}^{+ iw}\right)\right)}_{jj}}{\frac{1}{2\pi }{\int}_{-\pi}^{\pi }{\left(\varPsi \left({\omega}^{- iw}\right)\varSigma {\varPsi}^{\prime}\left({e}^{+ iw}\right)\right)}_{jj}d{\lambda}^{\prime }}$$

where the power of the jth variable is the aggregate of frequencies to a constant value of 2𝜋. This research employed Baruník and Krehlík (2018) model to refine the result of dynamic linkages obtained from D.Y. (2012) model. It helps investors to take investment decision in form of portfolio diversification in various time frequencies.

#### Wavelet analysis

Unlike Diebold and Yilmaz (2012) and Baruník and Krehlík (2018) models, wavelet analysis also captures the localized power variations among constituent variables. Generally, wavelet analysis consists of the continuous wavelet transform, cross wavelet transforms, and wavelet coherence. Wavelet coherence of wavelet analysis has been applied to refine the dynamic linkages or spillover in various time frequencies. The wavelet coherence examines the co-movement between time series in the time-frequency domain. It helps to detect the lead-lag relationship among variables, emphasizing frequency bands and time intervals (Torrence and Webster 1999). Mathematically, the coherence is presented as follows:

7 $${R}^2\left(u,s\right)=\frac{{\left|S\left({s}^{-1}{W}_{xy}\left(u,s\right)\right)\right|}^2}{S\left({s}^{-1}{\left|{W}_x\left(u,s\right)\right|}^2\right)S\left({s}^{-1}{\left|{W}_y\left(u,s\right)\right|}^2\right)}$$

where *S* is the smoothing operator with 0 ≤ *R*^2^(*u*, *s*) ≤ 1 with value 1 showing strong co-movement between time-series and vice-versa. The value of squared wavelet coherence measures the local linear correlation between stationary time series at each scale and is analogous to the squared correlation coefficient in linear regression. Although, the wavelet squared coherence analysis only considers positive values. Thus, it does not differentiate between positive and negative directions of the relationship between the time series. Additionally, a phase difference is introduced to provide information on the direction of the relationship and the causal relationship between time series. The same can be shown as below mathematically:

8 $${\phi}_{x,y}\left(u,s\right)={\tan}^{-1}\Big(\frac{\mathfrak{I}\left\{S\left({s}^{-1}{W}^{xy}\left(u,s\right)\right)\right\}}{\mathfrak{R}\left\{S\left({s}^{-1}{W}^{xy}\left(u,s\right)\right)\right\}}$$

where $\mathfrak{I}$ and $\mathfrak{R}$ are the imaginary and real part operators respectively. Graphically, the phase is represented by a black arrow on the wavelet coherence plots where a zero-phased difference implies co-movement among the series. One of the interesting facts of the model is that it represents the positive and negative connectedness with the help of arrows in graphical depiction; the left to right arrows implies the negative association while the right to the left indicates the positive association.

## Empirical results and discussion

### Diebold and Yilmaz (2012) followed by Baruník and Krehlík (2018)

Table 4 reports the results from Diebold and Yilmaz (2012), in which the matrix’s diagonal and off-diagonal elements are present within and cross-market spillovers or dynamic connectedness, respectively. Regarding the table, “From” in the last column shows the average value of linkages or connectedness obtained from other asset classes, whereas values in the sixth row “To” represent the average value of connectedness contributed to the other markets.

**Table 4:** Results derived from Diebold and Yilmaz (2012)

Series	RSPGB	RMGS	RIGW	RBTC	REEX	FROM
RSPGB	98.73	0.08	0.20	0.41	0.57	0.25
RMGS	0.75	89.35	9.52	0.24	0.14	2.13
RIGW	0.40	3.77	95.52	0.18	0.13	0.90
RBTC	0.34	0.06	0.06	99.53	0.01	0.09
REEX	0.58	0.01	0.15	0.02	99.24	0.15
TO	0.41	0.79	1.99	0.17	0.17	3.52
Net (From-To)	− 0.16	1.34	− 1.09	− 0.08	0.02	

It is revealed that RMGS has the highest connectedness (2.13) derived from other assets class, followed by RIGW (0.90), while RBTC has derived least connectedness (0.09). Furthermore, RIGW is the most contributor to the other assets class (1.99), followed by RMGS (0.79); the RBTC and REEX transmit the least value of shocks to other markets. Further, net directional connectedness helps to check whether an asset class transmits greater than it receives and vice versa. This study reveals the net transmitter or receiver of shocks among various asset classes considered (Tiwari et al. 2022). Referring to the estimates obtained in Table 4, it is observed that green bond (RSPGB), RIGB, and RBTC are net receivers of shocks with the values − 0.16, − 1.09, and − 0.08, respectively. It signifies that the MAC global solar energy index (RMGS) dominates the carbon and green market as it transmits more shocks (1.34) than other markets. Regarding the own-variable shocks depicted in Table 3, the research observed that 98.73% of the green bond, 89.35% of RMGS, 95.52% of RIGW, and 99.53% of RBTC, 99.24% of REEX are driven by their shocks within the behavior. It signifies that the index movement of RMGS by network connections is greater (10.65%) comparatively. Overall, it can be seen green bond is a net receiver of spillover and integrated marginally with other markets because of its uniquely mixed features of financial resources and environmental protection. Additionally, it reflects the transition toward a low-carbon transformation. Our results differ from the study of A.K Tiwari et al. (2022) and Broadstock et al. (2020) with respect to within shock and shock by network connection.

**Fig. 3:**
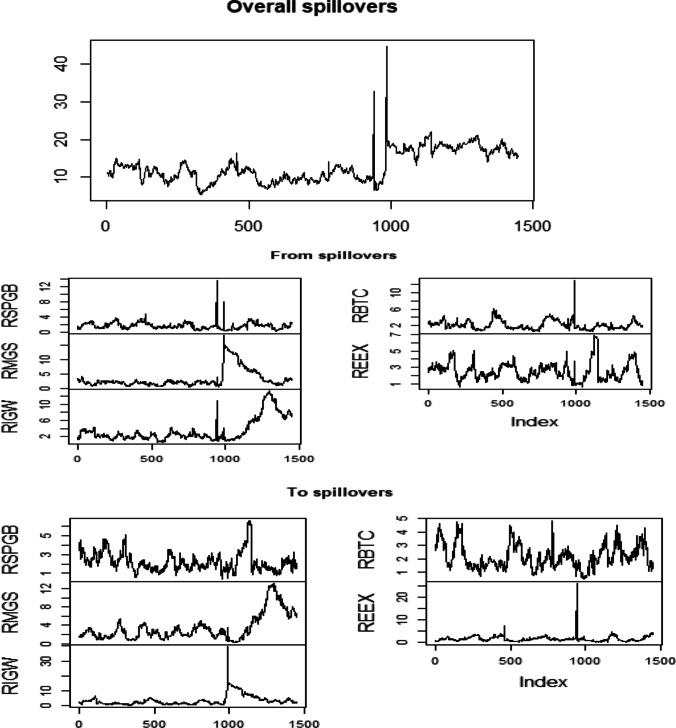
Graphical depiction of spillover using Diebold and Yilmaz (2012)

Further, overall spillover, from spillover to spillover using Diebold and Yilmaz (2012), has been shown in Fig. 3, respectively. In this figure, observations like 0, 500, 1000, and 1500 are equal to October 1, 2015, September 14, 2017, August 21, 2019, October 1, 2021, and December 13, 2021. The study reports that the lowest and highest overall connectedness is seen between the beginning of 2017 and August 2019. Further, spillover from the green bond, energy market (RMGS, RIGW), and bitcoin has a high network connection during 2019, nearly 12%. RIGW, followed by the carbon market, contributes large, considering the spillover contribution. The B.K. (2017) test was applied to examine the spillover from the green bond to the constituent assets class to refine the results.

In addition, Baruník and Krehlík (2018) model were also applied to examine the connectedness of green bond with energy, crypto, and carbon market. The B.K. (2017) test is presented in three different frequencies like short-term frequency connectedness (a day to 4 days), medium frequency connectedness (4 days to 10 days), and long-term frequency connectedness (10 days to infinity) in Tables 5, 6, and 7 respectively. In these consequent tables, “WTH” denotes within, “ABS” indicates the absolute, “From” represents the connectedness derived from other assets class, and “To” infers the spillover that has contributed to other indices. With respect to Table 5, it is observed that RMGS has the highest risk connectedness (0.90), followed by RIGW (0.69) obtained from other markets, whereas RIGW contributes high (0.80) followed by RMGS (0.60) in the short run. Moreover, RIGW, RBTS, and REEX are net contributors, while RSPGB and RMGS are net receivers of shocks in the short run. For the medium-run connectedness, RMGS is the highest receiver of shock (4.96), and RIGW is the highest contributor. The RIGW and RSPGB are net receivers, while the rest of the assets class are net receivers. Surprisingly, green bond (RSPGB) behaves differently in the medium run than short run as it is a net receiver. Regarding the long-term connectedness, RMGS remains the strong receiver, and RIGW is the highest contributor of shocks. Further, green bond (RSPGB), RIGW, and bitcoin (RBTC) are net contributors, whereas RMGS and REEX are net receivers. Our findings are similar to studies of Hanif et al. (2021) and Liu and Liu et al. (2021) while different from Tiwari et al. (2022) as they found that green bond is not the net contributor.

**Table 5:** B.K. test (2017)—roughly corresponds to 1 day to 4 days (band 3.14 to 0.79)

	RSPGB	RMGS	RIGW	RBTC	REEX	FROM_ABS	FROM_WITH
RSPGB	65.87	0.07	0.11	0.38	0.38	0.19	0.26
RMGS	0.22	61.03	2.71	0.21	0.14	0.66	0.90
RIGW	0.13	2.09	64.01	0.18	0.12	0.51	0.69
RBTC	0.23	0.03	0.03	75.69	0.01	0.06	0.08
REEX	0.32	0.00	0.08	0.02	90.88	0.09	0.12
TO_ABS	0.18	0.44	0.59	0.16	0.13	1.50	
TO_WTH	0.25	0.60	0.80	0.22	0.18		2.05
Net	0.01	0.30	− 0.11	− 0.14	− 0.06

**Table 6:** B.K. test (2017)—roughly corresponds to 4 days to 10 days (band 0.79 to 0.31)

	RSPGB	RMGS	RIGW	RBTC	REEX	FROM_ABS	FROM_WITH
RSPGB	20.18	0.00	0.06	0.02	0.12	0.04	0.24
RMGS	0.27	16.99	3.65	0.02	0.00	0.79	4.79
RIGW	0.14	1.00	18.93	0.00	0.00	0.23	1.39
RBTC	0.06	0.02	0.02	15.11	0.00	0.02	0.11
REEX	0.16	0.00	0.04	0.00	5.58	0.04	0.25
TO_ABS	0.13	0.20	0.75	0.01	0.02	1.12	
TO_WTH	0.77	1.24	4.58	0.05	0.15		6.78
Net	− 0.53	3.55	− 3.19	0.06	0.10

**Table 7:** B.K. test (2017)—roughly corresponds to 10 days to inf days (band 0.31 to 0)

	RSPGB	RMGS	RIGW	RBTC	REEX	FROM_ABS	FROM_WITH
RSPGB	12.68	0.00	0.03	0.01	0.07	0.02	0.22
RMGS	0.26	11.33	3.16	0.01	0.00	0.68	6.50
RIGW	0.12	0.68	12.58	0.00	0.00	0.16	1.53
RBTC	0.04	0.02	0.02	8.73	0.00	0.02	0.14
REEX	0.10	0.00	0.03	0.00	2.77	0.03	0.25
TO_ABS	0.11	0.14	0.65	0.00	0.01	0.91	
TO_WTH	1.00	1.33	6.15	0.03	0.14		8.65
Net	− 0.78	5.17	− 4.62	− 0.11	0.11		

It is documented that green bonds are marginally integrated with the energy and carbon market in risk transmission. This risk spillover differs in the short to long run; hence, the present research employs the B.K. (2017) test in three different time horizons. Risk decomposition in different periods plays a significant role for investors, arbitragers, traders, and speculators. Of course, the linkage of the two markets becomes different because of heterogeneous responses to the shocks. In this context, the overall diversification opportunity among green bonds, energy stock, bitcoin, and the carbon market is more in the short run than in the medium and long run as the total spillover is less in the short run; the same finding is found with the conclusion Tolliver et al. (2020).

**Fig. 4:**
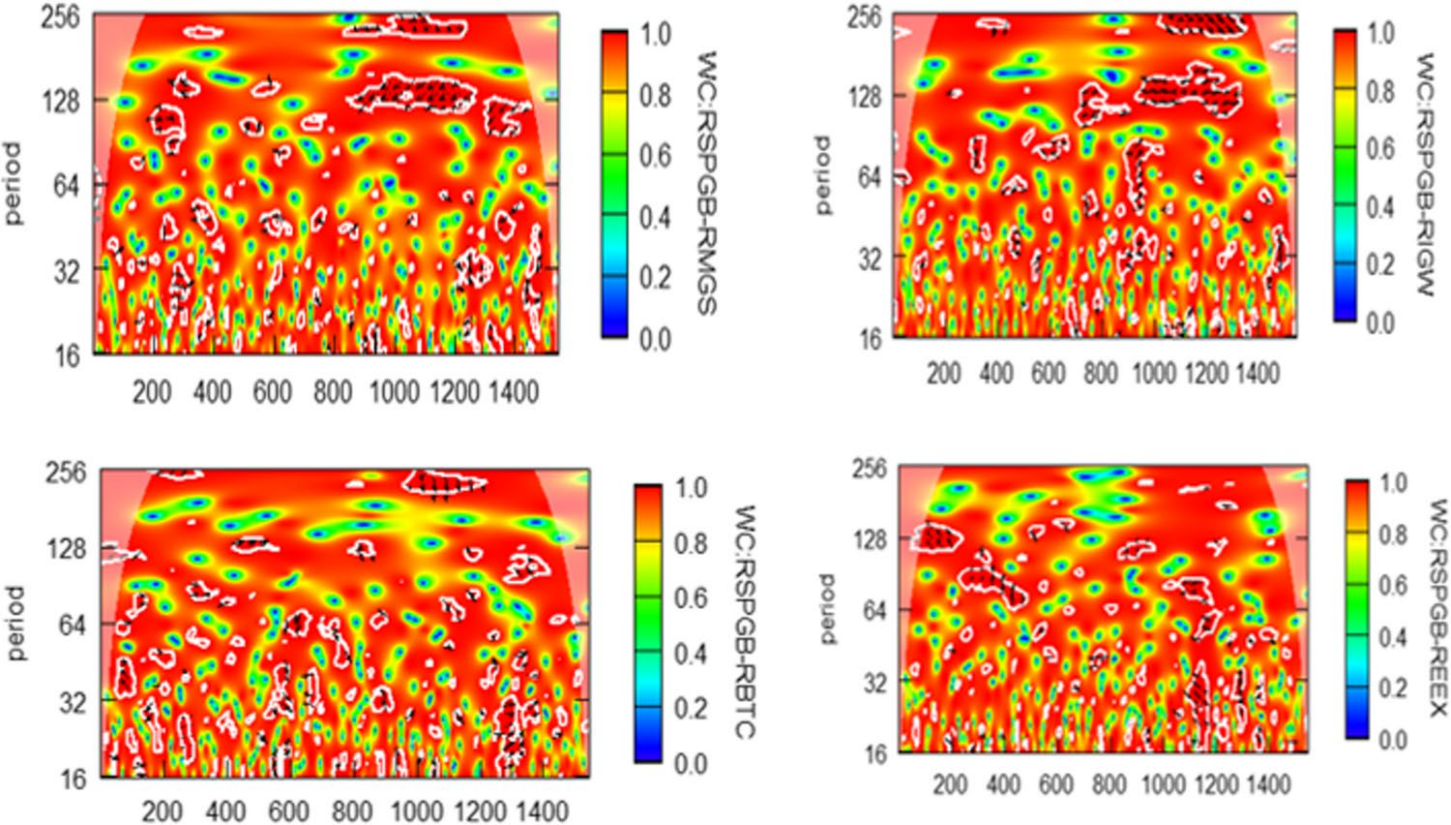
Wavelet coherence analysis

To refine the connectedness of green bond with constituent assets class, wavelet coherence analysis is displayed in Fig. 4. Unconditional correlation furnishes evidence of whether the various assets class are correlated or not, which spans a long period. Still, it does not depict and explore connectedness with frequencies over time. For the same, wavelet coherence is employed. It also helps to detect the lead-lag relationship among variables, emphasizing frequency bands and time intervals. The *Y*-axis and *X*-axis show the frequencies of scale and time, respectively. The time-frequency has been categorized into four cycles, namely short-scale (16–32 and 32–64 days), medium-scale (64–128 days), and long-scale (128–256 days). Similarly, the *X*-axis depicts the number of observations 200, 400, 600, 800, 1000, 1200, and 1400, which are on July 15, 2016, April 27, 2017, February 5, 2018, November 12, 2018, August 21, 2019, May 27, 2020, and March 3, 2021, respectively. For ease of interpretation (see Fig. 4), islands and arrows are presented in which red islands display strong coherence (near coefficient 1), whereas blue islands show weaker coherence (Liu and Liu 2020).

In the short run, there is no coherence from the green bond to the energy market (both RMGS and RIGW), crypto-market, and carbon market as the red and blue islands are scattered and not uniform. However, in the medium run (64–128 days), there is a correlation between the green bond and the energy market from the end of 2019 to mid-2020, as the red islands are present in significant areas. This might occur because of the COVID-19 outbreak; investors must consider these investment alternatives in their portfolios. Moving further for the long run (128–256), there is no association of green bond with the rest of the constituent assets class. Further, the movement of arrows from right to left or left to right is unclear as it is scattered and not uniform. Hence, there is no lead-lag relationship among these variables. Based on Diebold and Yilmaz (2012) and wavelet coherence, the marginal integration of the green bond with the rest of the constituent variables has been reported. The movement in a variable is caused by its own shock rather than by other variables’ shock. The findings agree with studies of Hanif et al. (2021) and Liu and Liu et al. (2021).

## Conclusion and policy implications

The new and expanding green bond market allows investors to engage in environmentally sound projects and diversify their portfolios to minimize risks (Abakah et al. 2021). In this context, one strives to seek connectedness among various investment alternatives they have. As there are burgeoning academic interests in the environment index like carbon emission, green bond, energy market, and technological (crypto) securities, we attempt to unravel the connectedness among these markets. The research considers the S&P green bond index (RSPGB) to measure the green bond to achieve our research objective. In contrast, MAC global solar energy index (RMGS) and ISE global wind energy index (RIGW) denote the energy market. In addition, to represent the cryptocurrency and carbon market, Bitcoin and the European energy exchange carbon index (REEX) are considered, respectively. The study deploys daily data of the markets mentioned above, extending from October 1, 2015 to December 13, 2021, and applies Diebold and Yilmaz (2012), Baruník and Krehlík (2018), and Wavelet coherence for the analysis. The study documents that green bond (RSPGB), energy market (RIGB), and Bitcoin (RBTC) are net receivers of shocks, and MAC global solar energy index (RMGS) dominates other markets as its transmission is high. The energy market (RMGS) has the highest risk connectedness in the short and medium run. Referring to the Baruník and Krehlík (2018) model, it is found that diversification opportunity exists in the short-run than medium and long-run as the total spillover is less in the short-run comparatively. The wavelet coherence produces a similar result as there is no coherence from the green bond to the energy market (both RMGS and RIGW) on a short and long scale. However, in the medium run (64–128 days), there is a correlation between the green bond and the energy market from the end of 2019 to mid-2020, as the red islands are present in significant areas. Additionally, the study also reports no lead-lag relationship among these markets.

Regarding dynamic spillover of green bond per se with constituent markets, the study documents various findings employing a battery of tests for the spillover like Diebold and Yilmaz (2012), Baruník and Krehlík (2018), and Wavelet coherence model. It is noticed that the energy market (RMGS) has the highest connectedness (2.13) derived from other assets class, followed by another index of the energy market (RIGW) which is 0.90. In contrast, bitcoin (RBTC) has derived the least connectedness (0.09). Overall, it can be seen that the green bond is a net receiver of spillover and integrated marginally with other markets because of its uniquely mixed features of financial resources and environmental protection. Focusing on dynamic connectedness in various time scales, it is found that one index of the energy market (RMGS) is the highest receiver of shock.

In contrast, another index of the energy market (RIGW) is the highest contributor in the short, medium, and long run, respectively. Additionally, the risk transmission is heterogeneous in different scales as the short period has less connectedness than the medium and long run. Hence, the overall diversification opportunity among the green bond, energy stock, Bitcoin, and carbon market is more in the short-run than in the medium and long run. Surprisingly, there is no lead-lag relationship among these markets. Badea and Pupazen (2021) have argued that bitcoin investments do not come under green investments and termed it “dirty currency.” It is also governed by black swan events and can provide hedge and significant diversification potential. These findings are similar to studies by Hanif et al. (2021), Liu and Liu et al. (2021), and Tolliver et al. (2020).

The findings of this study proffer the various implications. First, green bonds are less volatile than the rest of the constituent markets, allowing investors to use green bonds as investment assets while holding other asset classes. Second, as green bonds receive most shocks from the carbon market, policymakers can consider green bonds and carbon stocks as interrelated assets class in their policy framework. Similarly, it may furnish a portfolio composition containing these stocks for portfolio diversification. Third, investors can focus on portfolio hedges containing the energy market as one of the energy markets indexes (MAC global solar energy index-RMGS) is the highest receiver of shocks.

In contrast, another (ISE global wind energy index-RIGW) is the highest contributor to shocks. Fourth, due to the less connectedness of bitcoin with other markets, investors must park their money in bitcoin as it acts as a safe haven. Additionally, our findings on the dynamic connectedness of the green bond can assist in enacting the policies that assure a smooth recovery process in various market conditions.

Our findings propose that a study can be carried out to examine the dynamic connectedness employing the BEKK model, dynamic conditional correlation, quantile VAR, TPV-VAR, and asymmetrical connectedness among various markets. Based on the transmission of information, one can hold optimal portfolio weights and hedge ratios in various time duration like pre-Covid and post-Covid.

## Data Availability

All data generated or analyzed during this study can be available from corresponding author on request.

## Abbreviations

RSPGB: Return on S&P green bond index
RMGS: Return on MAC global solar energy index
RIGW: Return on ISE global wind energy index
RBT: Return on bitcoin
REEX: European Energy Exchange Carbon index
CO2: Carbon emission
GARCH: Generalized autoregressive conditional heteroscedasticity
DCC: Dynamic conditional correlation
TGARCH: Threshold GARCH
ADF: Augmented Dickey-Fuller
PP: Philips and Perron
LASSO: Least absolute shrinkage and selection operator
TVP-VAR: Time-varying parameter vector autoregressions

## Footnotes

1 https://www.statista.com/statistics/377382/bitcoin-market-capitalization/
